# Therapeutic relevance of the PP2A-B55 inhibitory kinase MASTL/Greatwall in breast cancer

**DOI:** 10.1038/s41418-017-0024-0

**Published:** 2017-12-11

**Authors:** Mónica Álvarez-Fernández, María Sanz-Flores, Belén Sanz-Castillo, María Salazar-Roa, David Partida, Elisabet Zapatero-Solana, H. Raza Ali, Eusebio Manchado, Scott Lowe, Todd VanArsdale, David Shields, Carlos Caldas, Miguel Quintela-Fandino, Marcos Malumbres

**Affiliations:** 1 Cell Division and Cancer group, Spanish National Cancer Research Centre (CNIO), Madrid, Spain; 2 Cancer Research UK Cambridge Institute, University of Cambridge, Cambridge, UK; 3 Memorial Sloan-Kettering Cancer Center, New York, USA; 4 Oncology R&D Group, Pfizer Worldwide Research & Development, Pfizer Inc., New York, USA; 5 Breast Cancer Clinical Research Unit, CNIO, Madrid, Spain

## Abstract

PP2A is a major tumor suppressor whose inactivation is frequently found in a wide spectrum of human tumors. In particular, deletion or epigenetic silencing of genes encoding the B55 family of PP2A regulatory subunits is a common feature of breast cancer cells. A key player in the regulation of PP2A/B55 phosphatase complexes is the cell cycle kinase MASTL (also known as Greatwall). During cell division, inhibition of PP2A-B55 by MASTL is required to maintain the mitotic state, whereas inactivation of MASTL and PP2A reactivation is required for mitotic exit. Despite its critical role in cell cycle progression in multiple organisms, its relevance as a therapeutic target in human cancer and its dependence of PP2A activity is mostly unknown. Here we show that MASTL overexpression predicts poor survival and shows prognostic value in breast cancer patients. MASTL knockdown or knockout using RNA interference or CRISPR/Cas9 systems impairs proliferation of a subset of breast cancer cells. The proliferative function of MASTL in these tumor cells requires its kinase activity and the presence of PP2A-B55 complexes. By using a new inducible CRISPR/Cas9 system in breast cancer cells, we show that genetic ablation of *MASTL* displays a significant therapeutic effect in vivo. All together, these data suggest that the PP2A inhibitory kinase MASTL may have both prognostic and therapeutic value in human breast cancer.

## Introduction

Protein phosphatase 2A (PP2A) is the major serine-threonine phosphatase in mammals. PP2A function as a multimeric complex containing a catalytic (C), scaffold (A) and regulatory (B) subunit. Regulatory subunits can belong to four different subfamilies: B (PR55), B′ (B56 or PR61), B″ (PR72), and B‴ (PR93/PR110), each one composed of multiple isoforms. Aberrant expression, mutations and somatic alterations of the PP2A scaffold and regulatory subunits have been detected in several types of human cancer [1, 2]. In particular, deletions in *PPP2R2A*, the gene encoding the α isoform of the PP2A regulatory subunit B55, are amongst the most frequent events in luminal-like breast cancer and define a sub-group of aggressive tumors [3, 4]. *PPP2R2B*, encoding B55β, is frequently inactivated by methylation in breast tumors [5] and a genetic variant in this gene associates with altered breast cancer risk and recurrence [6], thus suggesting that PP2A-B55 complexes may play a tumor suppressor role in breast cancer.

During the last years, the cell cycle kinase MASTL (also known as Greatwall) has emerged as a key player in the regulation of PP2A phosphatase during mitosis. MASTL was originally identified in *Drosophila* as a protein required for DNA condensation and normal progression through mitosis [7]. MASTL phosphorylates two small proteins, endosulfine (ENSA) and ARPP19, which in their phosphorylated form bind and inhibit PP2A-B55 complexes [8–11]. In vertebrates, PP2A-B55 complexes counteract the phosphorylation of CDK substrates [12]. The inhibitory function of MASTL over PP2A is required to maintain the mitotic state, whereas inhibition of MASTL and reactivation of PP2A is required for mitotic exit [13–16].

Although the function of MASTL during mitosis has been deeply characterized in multiple organisms [7], our understanding of its relevance in human cancer is still limited. Recent data suggest that MASTL may promote cell transformation in an ENSA/PP2A-independent manner by hyperactivating AKT [17]. MASTL is overexpressed in specific tumors such as oral squamous cell carcinoma, colon cancer and neuroblastoma [17–19] and data from knockdown screens suggest its therapeutic value in thyroid tumor cells [20, 21]. Mastl is also involved in recovery from DNA damage [22, 23] and its downregulation may therefore sensitize cancer cells to radiotherapy [18, 24].

In this work, we focus on the relevance of MASTL kinase activity in breast cancer, a tumor type in which its downstream target, PP2A-B55, has been suggested to be important for cancer progression. Here we show that *MASTL* knockdown or knockout using RNA interference or inducible CRISPR/Cas9 models results in impaired proliferation of some breast cancer cell lines. Sensitive cancer cells require MASTL kinase activity and expression of the B55 subunits of PP2A, suggesting the presence of a subgroup of breast cancer patients that could benefit from MASTL-directed therapies. Moreover, elevated levels of MASTL protein correlate with poor disease outcome, and may have prognostic value in Estrogen Receptor (ER)-positive breast tumors independently of the Ki67 proliferation marker.

## Results

### MASTL depletion differentially affects proliferation in breast cancer cell lines

**Fig. 1:**
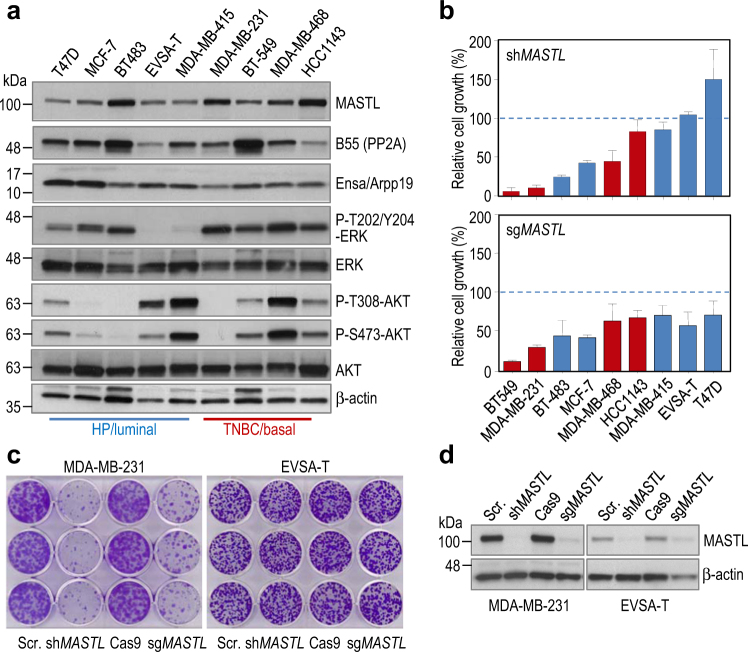
Effect of MASTL knockdown and knockout in breast cancer cell lines **a** Immunoblotting analysis of MASTL protein abundance in asynchronous luminal- and basal-like cancer cell lines. Protein levels for ENSA/ARPP19 and the B55 family of PP2A regulatory subunits were detected with pan-antibodies recognizing both ENSA and ARPP19, and different B55 isoforms, respectively. Activation of ERK and AKT oncogenic pathways was analyzed with the indicated antibodies. β-actin was used as a loading control. **b** Effect of shRNA-mediated MASTL knockdown (upper panel) or CRISPR/Cas9-mediated knockout (bottom panel) in the indicated breast cancer cell lines. Quantification of cell growth based on clonogenic assays is shown. For each cell line, cell growth of cells infected with a scramble-shRNA vector (upper panel) or an empty vector expressing only Cas9 (lower panel) was set as 100%. Bars indicate means + SD for at least three independent experiments. **c** Crystal violet staining of a clonogenic assay in representative examples of sensitive (MDA-MB-231) and resistant (EVSA-T) cell lines. **d** Western-blot analysis of MASTL protein levels after knockdown (shMASTL) or knockout (sgMASTL) in the two representative cell lines. Whole-cell lysates were obtained 5 days after lentiviral infection. *HP* hormone-positive, *TNBC* triple-negative breast cancer

Given the potential relevance of MASTL-PP2A/B55 pathway in breast cancer, we first analyzed MASTL expression and the consequences of its depletion in a panel of breast tumor cell lines, including both hormone-positive/luminal (T47D, MCF-7, BT-483, EVSA-T, MDA-MB-415) and triple-negative/basal-like (MDA-MB-231, BT-549, MDA-MB-468, HCC1143) subtypes. MASTL was differentially expressed in these cell lines without an obvious correlation with the estrogen receptor status or the primary oncogenic events present in these cells (Fig. 1a). We then used different short hairpin RNAs (shRNAs) to knock down *MASTL* expression in these cells. Several sequences including shRNAs #1, 3, 5, 6, 8, and 10 (Supplementary Fig. S1 and Supplementary Table S1) resulted in a significant downregulation of *MASTL* expression. However, we realized that some of these sequences provoked different phenotypes (data not shown) and decided to test their specificity in rescue assays in which a mouse Mastl cDNA (which is insensitive to these shRNAs) was ectopically expressed. As shown in Supplementary Fig. S1, only the growth defects caused by sh*MASTL* #8 were fully rescued by Mastl re-expression, suggesting common off-target effects after MASTL knockdown by RNAi means. We therefore decided to use sequence #8 in the rest of the assays.

As an alternative strategy we also decided to use the CRISPR/Cas9 system to induce genetic disruption of the *MASTL* gene. Three different small guide RNAs (sgRNAs) were tested and sgRNA #2 was selected for further studies (Supplementary Fig. S1).

Parallel knock down (shRNA #8; sh*MASTL*) and knock out (sgRNA #2; sg*MASTL*) assays elicited differential responses to MASTL depletion after lentiviral infection of a cell line panel with these sequences. As shown in Fig. 1b, some cell lines such as MDA-MB-231 or BT-549 were highly sensitive to *MASTL* knock down, whereas other cell lines such as EVSA-T or T47D were largely insensitive, despite showing similar level of *MASTL* depletion (Fig. 1c, d, and Supplementary Fig. S2). A similar trend was observed in *MASTL* knock out assays using the CRISPR reagents, although the differences in sensitivity to *MASTL* depletion were not so dramatic in this case (Fig. 1b), perhaps as a consequence of the complete elimination of MASTL due to genetic knockout in this technique. Both knock-down and knock-out of *MASTL* in the cell line MCF10-A also impaired proliferation of these non-transformed mammary epithelial cells (Supplementary Fig. S2). Interestingly, surviving clones in the resistant cell lines expressed low levels of MASTL, possibly as a consequence of in-frame indels at the genomic level (Supplementary Fig. S3). These data suggest that complete elimination of MASTL may be lethal in both sensitive and relatively insensitive cell lines, but much reduced levels of this kinase are sufficient to sustain cell proliferation in resistant cells.

We did not find any obvious correlation between the effect of *MASTL* depletion and the expression levels of MASTL or its substrate ENSA among the different tumor cell lines (Fig. 1a). Both sensitive and insensitive cells displayed a significant reduction in the phosphorylation of ENSA (Supplementary Fig. S2). However, cells with low expression of B55 subunits, such as EVSA-T or HCC1143, were among the least affected by *MASTL* silencing (Fig. 1a, b).

### Inducible MASTL knock out results in cell cycle defects in a kinase-dependent manner

**Fig. 2:**
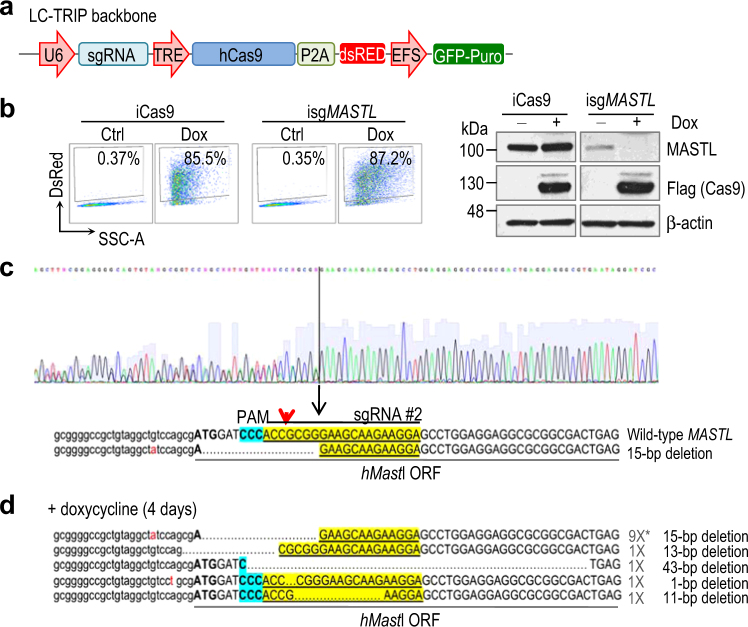
Inducible genetic ablation of MASTL using CRISPR/Cas9 techniques in breast cancer cells **a** Schematic representation of the sgRNA/cas9- inducible lentiviral vector used in this study. **b** Representative flow cytometry plots of dsRed induction in MDA-MB-231 cells expressing both Cas9 and sgRNAs against MASTL (isg*MASTL*) or control cells expressing Cas9 alone (iCas9). Numbers indicate the percentage of DsRed-positive cells after 4 days treatment with doxycycline. Immunobloting analysis of MASTL protein levels in the absence or presence of doxycycline (4 days treatment) in the two MDA-MB-231 selected clones is shown on the right panel. Cas9 induction was monitored with an anti-Flag antibody. **c-d** Genomic analysis of isg*MASTL* clones. sgRNA and PAM sequence are highlighted in yellow and blue colors, respectively. Red arrowhead denotes predicted Cas9 cutting site. Note that this clone is already heterozygous for *MASTL* before treatment due to a 15 bp deletion that leads to a frameshift mutation and a premature stop codon **c**. Treatment with doxycycline results in deletion of MASTL in both alleles due to 1–43 bp deletions also causing frameshift mutations as indicated in panel d. Representative sequences are shown

In order to analyze in more detail the consequences of *MASTL* ablation in cancer cells, we generated a conditional CRISPR/Cas9 system in which *MASTL* genetic knock out could be achieved in an inducible manner. We subcloned the sgRNA #2 sequence in the LC-TRIP vector, which constitutively expresses a green fluorescent protein (GFP), and conditionally expresses Cas9 and a red fluorescent protein (dsRed) following treatment with doxycycline (Fig. 2a). Stable MDA-MB-231 selected clones carrying this construct showed a clear induction of dsRed after treatment with doxycycline, which correlated with induction of Cas9 expression (Fig. 2b). We selected one of these clones, MASTL_sg2#5 (referred to as isg*MASTL*), in which one of the MASTL alleles displayed a small deletion even in the absence of doxycycline, indicating some level of leakiness in the LC-TRIP vector (Fig. 2c). As previously reported in mouse cells [16], cells with only one allele of MASTL grew normally with no differences versus homozygous wild-type cells (data not shown). Treatment of the isg*MASTL* clone with doxycycline resulted in a variety of deletions in the remaining wild-type allele efficiently generating knockout cells (Fig. 2d), which resulted in the elimination of MASTL protein (Fig. 2b). No off-target effects were identified after sequencing top-ranking potential off-target genomic sequences (Supplementary Table S2). As a control for the potential effects of Cas9 overexpression and/or doxycycline treatments, we selected one clone, Cas9#1 (referred to as iCas9), which carries the empty vector and therefore also expresses Cas9 and the fluorescent reporter (dsRed) upon doxycycline addition (Fig. 2b).

**Fig. 3:**
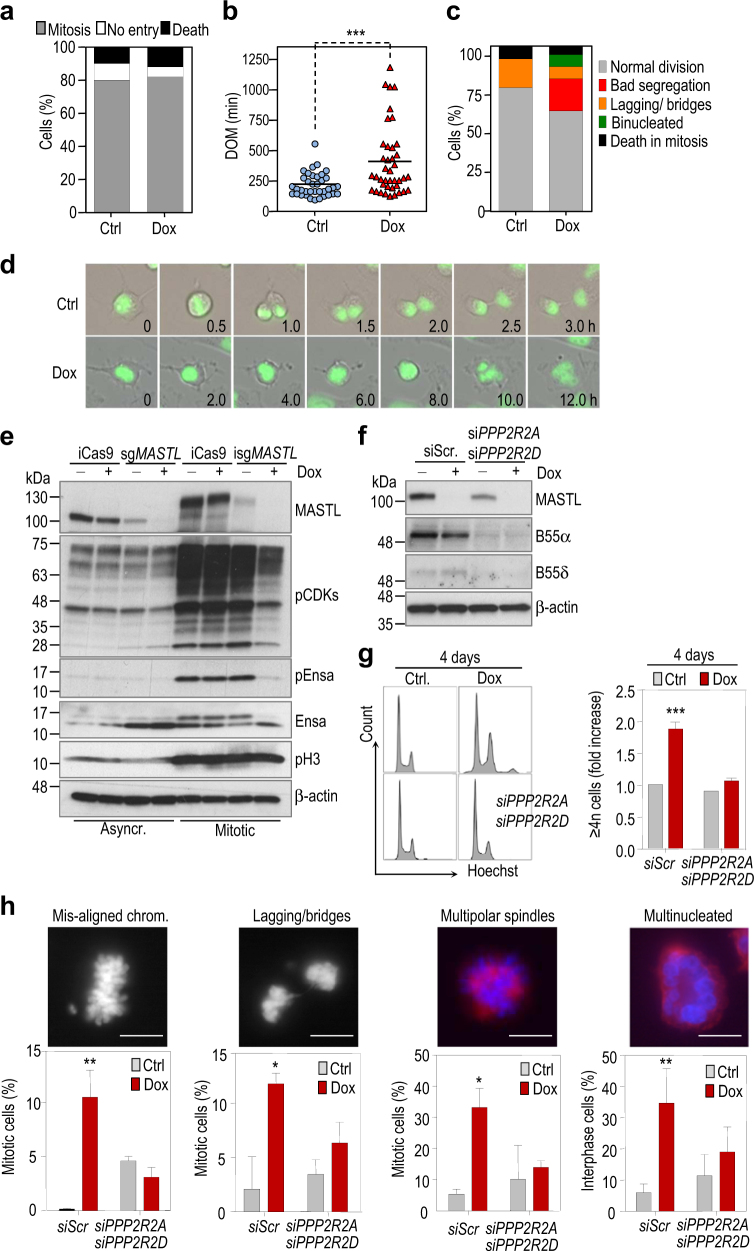
Cell cycle defects after MASTL knockout are B55-dependent **a-d** Mitosis was monitored by videomicroscopy in isg*MASTL* cells stably expressing H2B-GFP in the absence and presence of doxycycline. Video recording started 48 h after doxycycline administration. Graphs indicate the percentage of cells entering mitosis **a**, the duration of mitosis (DOM) from nuclear envelope breakdown until mitotic exit based on DNA decondensation and loss of rounded morphology **b**, and the classification of mitotic aberrations **c**. Representative time-lapse images are shown in **d**. ***, *p* < 0.001; Student *t* test. **e** Western-blot analysis of MASTL protein levels and phosphorylation and total levels of its substrate Ensa in the absence and presence of doxycycline (4 days treatment). Phosphorylation level of CDK substrates is detected with a pan-antibody against (K/R) pSPX (K/R) motifs. Phosphorylation of histone H3 is used as a marker of the mitotic state and β-actin is used as a loading control. Left lanes correspond to asynchronous-growing cells whereas right lanes represent nocodazole-enriched mitotic cells. Note that the band corresponding to MASTL is shifted in mitotic cell extracts due to its hyperphosphorylation state. **f** Immunoblotting analysis of B55α (*PPP2R2A*) and B55δ (*PPP2R2D*) PP2A subunits upon siRNA transfection in isg*MASTL* cells. **g** siRNA-mediated silencing of *PPP2R2A/D* PP2A subunits rescues accumulation of ≥4n cells in sg*MASTL* cells. Left panel shows FACs cell cycle profiles and the graph on the right shows the fold induction in the percentage of ≥4n cells upon doxycycline (Dox) treatment. Bars indicate means + SD for two independent experiments. **h** Immunofluoresce analysis in isg*MASTL* cells transfected with scramble or *PPP2R2A/D* siRNAs in the presence or absence of doxycycline (4 days treatment). The graphs show the quantification in percentage (mean + SD) of the defects detected in MASTL-depleted cells. At least 40 mitotic cells and 100 interphasic cells were counted in each condition. Representative pictures of the indicated phenotypes are shown. Scale bar, 1 µm. *Scr* scramble

MDA-MB-231 *MASTL* knock out cells did not show a defect in mitotic entry (Fig. 3a), but these null cells displayed a significant increase in the duration of mitosis (Fig. 3b) accompanied by abnormal chromosome segregation or lack of segregation resulting in tetraploid cells (Fig. 3c, d), in agreement with the mitotic defects reported in other mammalian cells [13, 14, 16, 25]. Genetic ablation of *MASTL* was accompanied by a significant defect in the mitotic phosphorylation of its substrate endosulfine (ENSA; Fig. 3e), a small protein that inhibits PP2A-B55 complexes, an event observed both in sensitive and resistant cells (Supplementary Fig. S2). *MASTL* ablation in MDA-MB-231 cells was also accompanied by a general reduction of mitotic phospho-CDK substrates known to be dephosphorylated in a PP2A-dependent manner. Phosphorylation of the mitotic marker phospho-histone H3 (pH3S10) did not change upon *MASTL* ablation indicating that these defects were not a consequence of decreased number of mitotic cells but rather a consequence of a putative PP2A-B55 re-activation upon *MASTL* knockout (Fig. 3e). In agreement with this hypothesis, knock down of B55α and B55δ, the two ubiquitous members of the B55 family, partially rescued the defects observed in *MASTL* knockout cells (Fig. 3f-h). Treatment with doxycycline led to an accumulation of 4n and ≥ 4n cells as a consequence of the chromosome segregation failure described above, which was partially prevented in the presence of reduced levels of PP2A-B55 phosphatase (Fig. 3f, g). In agreement with these results, depletion of B55 subunits rescued the presence of multipolar mitotic spindles and multinucleated cells in MASTL-depleted cells. Other mitotic defects induced by MASTL deletion, such as chromosome mis-alignments in metaphase or lagging chromosomes and DNA bridges during anaphase and telophase, were also partially rescued upon B55 α and δ knock down (Fig. 3h). These data suggest that the reduced proliferation induced by MASTL ablation is very likely the consequence of severe mitotic defects, mostly caused by PP2A-B55 hyper-activation.

**Fig. 4:**
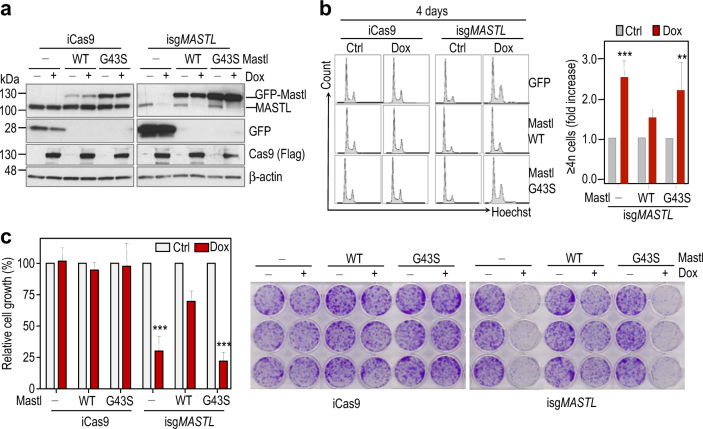
Breast cancer cells require MASTL kinase activity **a** Immunoblotting analysis of MASTL protein level in iCas9 and isg*MASTL* cells expressing murine wild-type (WT) or kinase dead (G43S) Mastl forms fused to GFP in the presence and absence of doxycycline (4 days treatment). **b** FACS profiles showing accumulation of ≥4n cells after depletion of MASTL and rescue by the WT, but not G43S, murine Mastl forms. The graph shows the fold induction in the percentage of ≥4n cells upon doxycycline (Dox) treatment. Bars indicate means + SD for three independent experiments. **c** Mastl WT, but not G43S, rescues cell growth in colony formation assays. Graph on the left panel shows cell growth quantification, where cell proliferation in the absence of doxycycline was set as 100%. Bars indicate means + SD for three independent experiments. The middle and right panels show the crystal violet staining of one representative experiment. ***, *p* < 0.001; Student *t* test. *Dox* doxycycline

We next stably transfected the inducible knock out MDA-MB-231 model with GFP-tagged wild-type or kinase-dead (G43S) murine *Mastl* sequences, which are insensitive to the sgRNA used in the CRISPR/Casp9 system (Fig. 4a). As described above, treatment with doxycycline induced an accumulation of 4n and ≥4n cells as a consequence of the mitotic defects generated upon *MASTL* ablation (Fig. 4b). These defects, as well as the reduced cell growth caused by MASTL knockout were significantly rescued by the wild-type, but not the kinase-dead, Mastl cDNA (Figs. 4b, c), suggesting that the therapeutic effect obtained upon MASTL elimination could also be achieved by inhibiting its kinase activity. All together, these results confirm the relevance of the ENSA-B55-PP2A pathway in the therapeutic effects achieved by elimination of MASTL kinase activity.

### *MASTL* ablation results in defective breast tumor growth in vivo

**Fig. 5:**
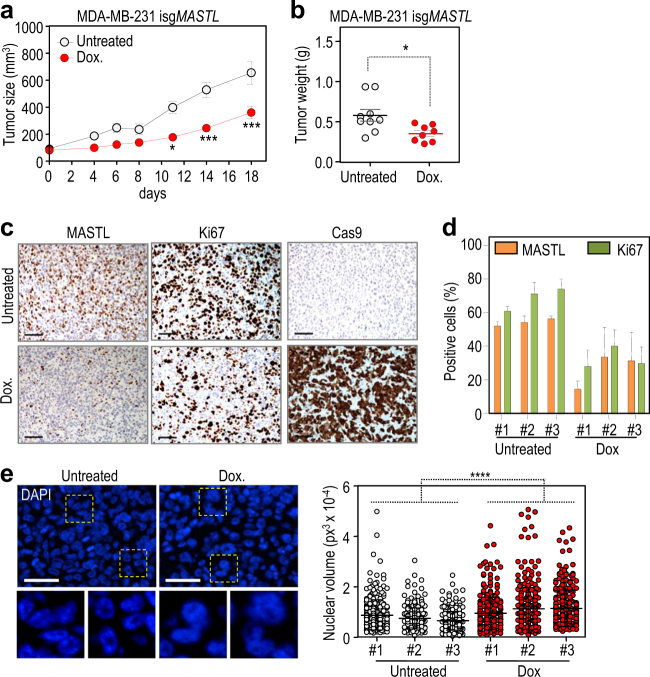
MASTL knockout impairs growth of tumor cells in vivo **a** Growth of isg*MASTL* MDA-MB-231 xenotransplants either untreated (white) or after continuous treatment with doxycycline-enriched diet (red). * *p* < 0.05, ***, *p* < 0.001; 2way ANOVA. **b** Tumor weight at the endpoint of the xenograft assay shown in **a**. * *p* < 0.05; Student *t* test. **c** Immunohistochemical analysis of MASTL, Ki67 and Cas9 protein levels in representative tumors of the isg*MASTL* xenograft assay. **d**. Histogram showing the percentage of MASTL- and Ki67-positive cells in three representative tumors of each experimental condition. Cas9-positive cells were scored in doxycycline-treated mice. Bars represent means + SD of three random areas per tumor. **e** Nuclear volume analysis in isg*MASTL* untreated and doxycycline-treated tumors at the endpoint. Representative images of DAPI staining are shown on the left panel. The graph shows the quantification of three representative tumors of each experimental condition (untreated and continuous doxycycline treatment). ****, *p* < 0.0001; Mann-Whitney test. *Dox* doxycycline. Scale bars, 25 µm

To analyze the effect of MASTL ablation on tumor growth we generated xenografts of the inducible MDA-MB-231 clones. Once these tumors reached 100 mm^3^, mice were treated with doxycycline-supplemented diet or kept untreated. We also included a third group of mice that received a transient treatment during the first week and doxycycline was removed afterwards. As represented in Figs. 5a, b, mice treated with doxycycline showed a significant reduction in tumor size and weight at the endpoint of the experiment. As an additional control, we also used MDA-MB-231 clones expressing the inducible Cas9 vector in the absence of the specific *MASTL* sgRNA. Treatment with doxycycline did not alter growth of these control tumors suggesting that neither Cas9 nor doxycycline itself had a significant effect in tumor growth (Supplementary Fig. S4). In the *MASTL* inducible knock out (isg*MASTL*) clone, transient treatment with doxycycline only for 1 week resulted in an initial reduction in tumor growth but this effect was lost after elimination of doxycycline (Supplementary Fig. S4). To discriminate whether this effect was due to partial efficiency by the inducible CRISPR/Cas9 model or to adaptation to the loss of MASTL, we quantified the number of MASTL-positive cells in these tumors. As showed in Fig. 5c, sustained treatment with doxycycline resulted in a significant induction of Cas9 and decrease in the number of cells expressing MASTL. However, the percentage of MASTL-expressing cells was significantly higher when doxycycline treatment was transient, suggesting that these tumors were repopulated with cells in which MASTL has not been knocked out (Supplementary Fig. S4). Given that the activity of the inducible CRISPR/Cas9 model is only partial, the difference in tumor growth between control and MASTL-null cells is possibly underestimated in these assays. Importantly, the reduction in the number of MASTL-positive cells upon doxycycline treatment correlated with a reduction in the number of proliferating cells, determined by Ki67 staining in Cas9-positive cells (Fig. 5d and Supplementary Fig. S4). Moreover, doxycycline-treated tumors frequently display aberrant nuclear morphologies and bigger nuclei, suggesting segregation failure due to MASTL ablation, as already observed in cultured cells in vitro (Fig. 5e). Collectively, the results obtained in these tumor models suggest that elimination of MASTL activity might be a valuable therapeutic target in specific breast tumors.

### MASTL overexpression correlates with poor disease outcome in human breast cancer

**Fig. 6:**
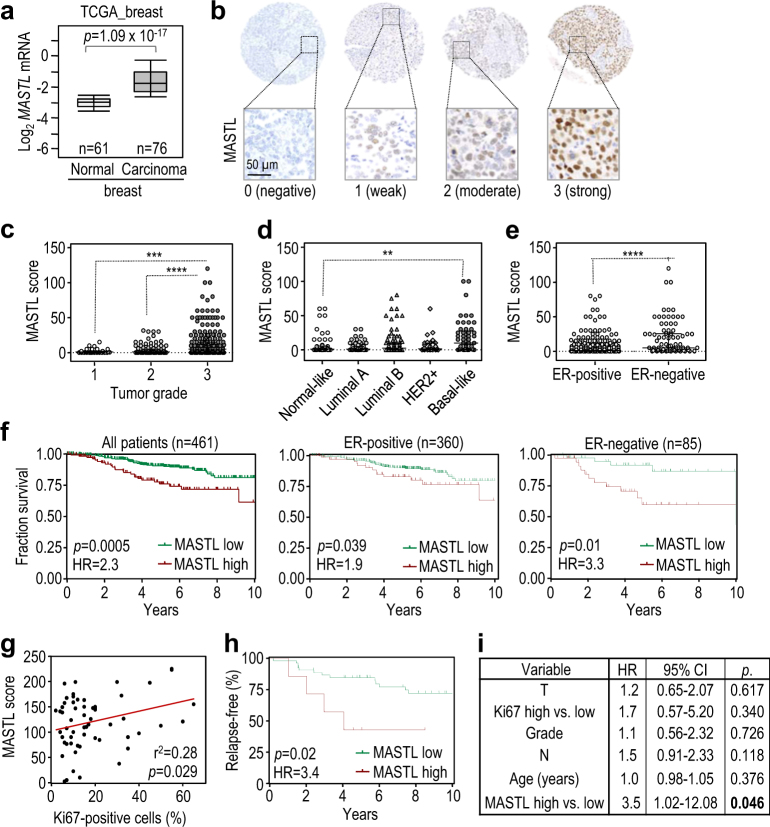
Expression of MASTL in human breast cancer **a** *MASTL* mRNA levels in breast carcinomas (*n* = 76) or normal breast tissues (*n* = 61) in the TCGA breast cancer cohort (Oncomine database). **b** Immunohistochemical detection of MASTL in breast cancer samples, showing null (0), weak (1), medium (2) or strong (3) levels of nuclear staining. **c-e** Correlation of MASTL protein expression levels with the pathological grade **c**, the molecular subtypes **d** and the ER status **e** of breast tumors from the METABRIC cohort. Black lines in each group indicate median with interquartile range. ** *p* < 0.01, ***, *p* < 0.001, ****, *p* < 0.0001; P values were calculated using the non-parametric Kruskal-Wallis test. **f** Kaplan-Meier analysis of survival, comparing breast cancer patients with high vs. low MASTL protein expression (METABRIC cohort). Statistical significance was calculated using the log-rank test. **g** Dot-plot diagram showing the correlation between MASTL protein expression and Ki67 levels (percentage of positive cells) in an independent cohort of hormone-positive breast cancer patients. Statistical analysis was performed using the Pearson’s test. **h** Kaplan-Meier plot for disease relapse, comparing high vs. low MASTL protein expression in the hormone-positive cohort of breast cancer patients. Statistical significance was calculated using the log-rank test. **i** Cox´s Proportionate Hazards Model, showing the risk variation attributable to each variable per unit increase. MASTL level is the only variable independently associated with an increased relapse risk. MASTL score (0-300) for **c**-**e** and **g** was calculated by multiplying the percentage of Mastl-positive cells by the intensity value of the staining according to the criteria shown in **b**. *ER* estrogen receptor, *HR* hazard ratio

Analysis of *MASTL* expression in human tumors showed increased levels of the *MASTL* mRNA in breast cancer compared to normal tissue, correlating inversely with recurrence-free survival in ER-positive breast tumors (Fig. 6a and Supplementary Fig. S5). To analyze the expression of this kinase at the protein level in human tissues we first generated a new monoclonal antibody against the human protein. This antibody identifies a single band in immunoblots that corresponds to the expected MASTL size and is suppressed by specific short hairpin RNAs against this kinase (Supplementary Fig. S5). MASTL is mostly nuclear in cancer cells as previously described [18] and its expression is highly variable among tumors (Fig. 6b). We next used this antibody to detect MASTL in three different cohorts of breast cancer samples totaling more than 500 patients.

The first cohort included 461 patients representing different subtypes of breast cancer from the METABRIC study. [3] MASTL abundance was significantly associated with higher histological grade (Fig. 6c) and was more evident in basal-like (Fig. 6d) and ER-negative (Fig. 6e) tumors. We also detected a positive correlation between MASTL protein expression and the mRNA levels of Ki67 (Spearman’s rho = 0.18, *p* = 0.0002) and Aurora A kinase (Spearman’s rho = 0.21, *p* = 0.0001), suggesting a possible link with cell proliferation. Importantly, high levels of MASTL associated with poor prognosis in this set of patients (hazard ratio (HR) = 2.3; 95% CI, 1.57–5.02; *p* = 0.0005), and more robustly in ER-negative (HR = 3.3; 95% CI, 1.4–10.5; *p* = 0.01) vs. ER-positive tumors (HR = 1.9; 95% CI, 1.04–4.3; *p* = 0.04; Fig. 6f).

We also analyzed MASTL expression in two other sets of breast cancer including hormone receptor-positive (*n* = 73; 12.5 years of follow-up) and triple-negative (TNBC; *n* = 84; 11.7 years of follow up] breast tumors (Supplementary Tables S3 and S4). MASTL levels also correlated with Ki67 expression (as scored by immunohistochemistry) both in hormone-positive tumors (Pearson’s R2 = 0.28; *p* = 0.029; (Fig. 6g) and TNBCs (Spearman’s rho = 0.396; *p* = 0.0003; Supplementary Fig. S5). Clinical data confirmed that MASTL overexpression correlated with poor disease outcome in hormone-positive tumors. Patients with high MASTL levels had a median time to relapse of 4.06 years vs. not reached for the remainder (average time: 5.5 vs. 10.8 years; HR = 3.4; 95% CI, 1.4–44.6; *p* = 0.02; Fig. 6h). The low magnitude of the correlations observed between MASTL and Ki67 suggests that MASTL might have an independent impact in the disease prognosis regardless of its potential functional implication in driving higher or lower replication indexes. Interestingly, when adjusted by the conventional prognostic factors, including high vs. low Ki67 using 14% as a cut-off point, high MASTL levels were highly and strongly associated with poor clinical outcome, conferring a >3.5-fold increase in the risk of relapse (Fig. 6i), suggesting a prognostic role independent of the Ki67 fraction. A similar trend was found in the TNBC cohort, although it did not reach statistical significance (Supplementary Fig. S5). These data suggest that, besides its potential as a new druggable therapeutic target, MASTL may also have a significant prognostic value in breast cancer.

## Discussion

During recent years, MASTL has attracted significant interest as a kinase module that can inhibit major cellular phosphatases during mitosis. [7] The ability of MASTL to phosphorylate and activate small PP2A inhibitors has opened a new paradigm in the direct control of phosphatases, and in particular PP2A-B55 complexes, by kinases during cell cycle progression [10, 11]. PP2A is a major tumor suppressor whose activity is inhibited by different means in human cancer. In addition to mutations in the scaffold subunits, B55α (encoded by the *PPP2R2A* gene) and B55β (encoded by *PPP2R2B*) subunits are occasionally eliminated by deletion or hypermethylation of the corresponding genes [1, 3, 5] suggesting the relevance of PP2A-B55 activity in preventing breast cancer development. We therefore hypothesized that MASTL activity could also contribute to breast cancer progression through the inactivation of these complexes.

Elimination of MASTL by RNA interference or CRISPR/Cas9 technology prevents proliferation of certain luminal- and basal-like breast tumor cell lines without a clear correlation with specific histological subtypes. Previous work suggested that exogenous overexpression of MASTL may further trigger AKT activation in an ENSA/PP2A independent manner [17]. However, we did not find any correlation between the protein levels of MASTL and the extent of AKT phosphorylation in the panel of breast cell lines used in this study. Importantly, our data indicates that downregulation of endogenous MASTL in these cells, either using RNA interference or CRISPR/Cas9 means, results in mitotic defects accompanied by reduced phosphorylation of ENSA and CDK substrates. In line with these observations, these defects can be rescued upon downregulation of B55 subunits suggesting that the therapeutic effect of targeting MASTL is at least partially due to its ability to inhibit PP2A-B55 complexes and that tumor cells with inactivation of B55 are likely resistant to this therapeutic effect. None of the proliferation defects caused by MASTL depletion could be rescued by a kinase-deficient mutant, demonstrating the requirement of MASTL catalytic activity for proliferation of breast tumor cells. Since MASTL is a druggable target, this is especially relevant for the development of small-molecule inhibitors and their potential therapeutic use in the future. Besides the presence of its target B55, it remains to be determined what the other requirements for sensitivity to MASTL depletion are. A significant number of breast tumor cell lines were resistant to MASTL depletion despite expressing normal levels of their targets, ENSA and B55, suggesting unknown mechanisms of resistance.

In vivo, overexpression of MASTL contributes to the growth of certain tumor cell lines in xenograft assays, whereas cells with MASTL knockdown display reduced growth in these assays [17]. Yet, the therapeutic effect of MASTL depletion in established tumors was not previously tested. Using a new inducible CRISPR/Cas9 model in vivo we have shown that ablation of MASTL in tumors results in impaired tumor progression (Fig. 5). The results from this assay likely underestimate the effect of MASTL ablation since a significant percentage of tumor cells maintain MASTL expression as a technical limitation of this genetic model. Yet, these results suggest that MASTL inhibition may contribute to the reactivation of PP2A in specific tumor types, an attractive possibility given the relevance of PP2A activation as a therapeutic strategy in cancer [26].

Besides the relevance of MASTL as a potential new therapeutic target for breast cancer, our data suggest it may also have prognostic value in breast tumors. Overexpression of MASTL at the protein level has been reported in several tumor types [17–19], yet its correlation with disease outcome was not deeply explored. Our data on breast cancer patients indicates that high levels of MASTL protein correlates with tumor aggressiveness and predicts poor survival in two independent cohorts of ER + tumors, in line with the conclusions from a recent report using mRNA data [27]. In hormone-positive breast cancer, Ki67 is currently the most important prognostic factor when adjusted by tumor size, nodal status, or grade [28]. However this association is usually evident in large patient series because the atributtable risk to a high Ki67 fraction is relatively low. A multivariate analysis, including high vs. low Ki67, showed that high levels of MASTL constitute a Ki67-independent risk factor for relapse in hormone-positive tumors. Interestingly, we also found a strong association between MASTL overexpression and poor prognosis in ER- tumors, a subtype where Ki67 did not work as a prognostic factor. However, this association was not detected at the mRNA level (Supplementary Fig. S4 and Ref. [27], perhaps reflecting either different mechanisms of regulation of MASTL expression at the mRNA vs. protein level in ER- tumors compared to ER+ ones, or heterogeneity between the different patient cohorts used for mRNA versus protein studies. Although new studies are required to understand resistance mechanisms or possible synergies with other therapeutic strategies, MASTL kinase emerges not only as a new therapeutic target, but also as a potential biomarker with prognostic value in breast cancer.

## Materials and methods

### Cell culture, drugs and cell proliferation analysis

All cancer cell lines were obtained from American Type Culture Collection, and were maintained in DMEM or RPMI-1640 medium supplemented with 10% fetal bovine serum. The MDA-MB-231 cell line was authenticated by short tandem repeat profiling, using the GenePrint 10 System (Promega). The identity of the other cells has not been re-analyzed during the last 2 years. Mitotic cell extracts were obtained by treating cells with nocodazole (0.8 µM) during 14–16 h, and collecting cells by mitotic shake-off. For cell proliferation assays, cells were seeded in 12-well plates at low confluence (1-2 × 10^3^ cells) and fixed 10–15 days later with methanol. Colonies were stained with crystal violet (0.1% wt/vol), washed extensively, and imaged with a flatbed scanner. Quantification was performed by using a colony Area Image J Plugin [29].

### RNA interference and genetic editing by CRISPR/Cas9 systems

Silencing of MASTL was performed using pLKO.1 lentiviral plasmids encoding specific shRNA sequences and purchased from Sigma (Supplementary Table S1). To knock down *PPP2R2A* and *PPP2R2D* transcripts, specific siRNAs were purchased from Qiagen and transfected using Hiperfect (Qiagen), according to manufacturer’s instructions.

Genome editing of *MASTL* was performed with the CRISPR/Cas9 system. PX459 and lentiCRISPRv2.0 backbone vectors were obtained from Addgene (plasmids 48139 and 52961, respectively). LC-TRIP (U6-sgRNA-TRE-Cas9-P2A-dsRED-EFSGFP/Puro) lentiviral vector was constructed based on the lentiCRISPR backbone [30]. Using standard cloning techniques, the P2A-Puro was replaced by a P2A-dsRED with a 3′ multicloning site and inserted stepwise the EFS promoter and a GFP-Puro fused cassette downstream of the dsRED marker. In a last step, the EFS promoter driving the expression of hCas9 was replaced by the TRE promoter. All fragments were PCR-amplified from TRMPVIR vector [31]. sgRNAs targeting *MASTL* were designed according to available algorithms (http://crispr.mit.edu/) and subcloned into the pX459, lentiCRISPRv2 and pLC-TRIP vectors. Indel mutations were assessed by the T7E1 assay [32]. To generate iCas9 and isg*MASTL* inducible cell lines, MDA-MB-231 cells were infected with an rtTA-expressing retrovirus and selected with puromycin (1 µg/ml). MDA-MB231-rtTA cells were then infected with lentiviral LC-TRIP plasmids encoding specific sgRNAs against *MASTL*, sorted for GFP-positive cells and plated for single cell cloning. The expression of Cas9 was induced by adding 2 µg/ml doxycycline to the culture medium for 4 days. To exclude potential off-target effects of sgMASTL, we identified top-ranking off-target genomic sites (Supplementary Table S2) in the human genome, using a published prediction tool [33].

For rescue assays, the *Mastl* kinase-dead mutant (G43S) was generated by site-directed mutagenesis using mouse *Mastl* cDNA as a template. Both wild-type and the G43S mutant were subcloned as GFP fusions into the pLVXpuro lentiviral vector (Clontech).

### Antibodies and immunodetection

Mouse monoclonal antibody to human MASTL (available from Abcam, Riply 74C) was generated against recombinant full-length protein corresponding to amino acids 1-879 of human MASTL fused to a GST tag. A specific rabbit polyclonal antibody against the B55α PP2A subunit was generated by immunization with a synthetic peptide common to the human and mouse sequence (MAGAGGGNDIQWCFS, Genscript).

For histological analysis tissues were fixed in 10%-buffered formalin (Sigma) and embedded in paraffin wax. Sections of 3- or 5-μm thickness were stained with haematoxylin and eosin. Additional immunohistochemical examination was performed using specific antibodies against Ki67 (Master Diagnostica), Cas9 (Cell Signaling) and MASTL. The anti-MASTL antibody above described was used for human samples, whereas anti-Mastl 4F9 (Millipore) antibody was used in mouse samples. For immunofluorescence, cells were plated on coverslips, fixed with 4% formaldehyde, permeabilized in 0.5% Triton X-100, and stained with DAPI and an antibody against α-tubulin (Sigma). For immunoblotting cells were lysed in Laemmli buffer. Proteins were separated on TGX Criterion Bis-Tris acrylamide gels (BioRad), transferred to nitrocellulose membranes (BioRad), and probed using the following specific antibodies: phospho-ENSA (Ser67)/ARPP19 (Ser62), ENSA, phospho-p44/42 MAPK (Erk1/2) (Thr202/Tyr204), p44/42 MAPK (Erk1/2), phospho-Akt (Thr308), phospho-Akt (Ser473), Akt, and phospho-(Ser) CDKs Substrate from Cell Signaling; β-actin and Flag from Sigma; GFP from Roche; phospho-Histone H3 (Ser10) from Millipore; PP2A-B55-α (2G9) from Santa Cruz Biotechnologies; PPP2R2D (N2C3) from GeneTex; and MASTL and B55α antibodies generated in this study.

### Flow-cytometry

Flow cytometry analysis of DNA content was performed by cell fixation with cold 70% Ethanol followed by staining with 10 µg/ml Propidium Iodide (Sigma) or 10 μg/ml Hoechst 3342 (Molecular Probes, Thermofisher). Data acquisition was performed with a LSR Fortessa analyzer (BD Biosciences).

### Time-lapse microscopy

Cells were plated on eight-well glass-bottom dishes (Ibidi) and imaged with a Deltavision RT imaging system (Olympus IX70/71, Applied Precision) equipped with a Plan Apochromatic 20X/1.42 N.A. objective lens, and maintained at 37 °C in a humidified CO2 chamber. Images were acquired every 10 min, and analysis was performed using ImageJ software.

### Tumor mouse models

For xenograft tumor assays, athymic nude mice (7-week-old females provided by Harlan Laboratories/ENVIGO), were injected subcutaneously in both flanks with 7 × 10^6^ cells corresponding to the indicated MDA-MB-231 inducible clones (iCas9 or isg*MASTL*). Two weeks after injection, when tumors reached 100 mm^3^, mice were fed with doxycycline-supplemented diet (2000 mg/Kg, Research Diets Inc.). For each clone, 18 mice were divided in 3 experimental groups (6 mice/group): one group was maintained with standard diet, a second group was fed with doxycycline-supplemented diet during 2 weeks, and a third group was also treated with doxycycline but only during one week and then was switched back to standard diet (transient induction). Tumors volumes were monitored using caliper measurements. Mouse studies described in this manuscript have been approved by the Institutional Animal Care and Use Committee of the FIS-Comunidad de Madrid.

### Analysis of human tumors

Breast tumor samples from the METABRIC (Molecular Taxonomy of Breast Cancer International Consortium) study have been previously described. [3] Tumor samples from the hormone-positive and triple negative breast cancer cohorts were examined for tumor content by an expert pathologist, and two 3-mm cores were selected from cellular-rich regions, captured and randomly spotted into a tissue microarray. The study protocol was reviewed and approved by the Institutional Review Board at the Hospital 12 de Octubre (approval number 11/137).

An H-score (from 0 to 300) was generated for MASTL staining by multiplying the percentage of MASTL positive cells by the intensity score (0–3). The cut-off for high MASTL expression was established in the mean value plus one standard deviation, when the H-score showed a normal distribution. In the cohorts where the H-score showed a non-Gaussian distribution, the cut-off was established in the upper quartile. Kaplan Meier estimates and log-rank tests were used for studying the univariate impact of MASTL staining. Cox regression was used to determine the adjusted prognostic impact in disease relapse of MASTL levels, adjusting by the conventional prognostic variables.

### Statistical analysis

Statistical analysis was carried out using Prism 6 (Graphpad Software Inc.). All statistical tests of comparative data were done using two-sided, unpaired Student’s *t*-tests or ANOVA for differential comparison between two groups or more groups, respectively. In case of non-Gaussian distributions, Mann-Whitney and Kruskal-Wallis tests were used for comparison between two or more groups, respectively. Correlation analysis was performed using Pearson’s test or, alternatively, Spearman’s test for non-normal distributions. Data with *p* < 0.05 were considered statistically significant (*, *p* < 0.05; **, *p* < 0.01; ***, *p* < 0.001; ****, *p* < 0.0001).

## Electronic supplementary material

Supplementary Figures and Tables
