# Supplementary material for: Therapeutic relevance of the PP2A-B55 inhibitory kinase MASTL/Greatwall in breast cancer
Source: Cell Death Differ. 2017 Dec 11;25(5):828–40. doi: 10.1038/s41418-017-0024-0 (PMC5943447; doi:10.1038/s41418-017-0024-0)
Supplement: Supplementary file 1 — Supplementary Figures and Tables [file 41418_2017_24_MOESM1_ESM.pdf]

Supplementary Material

Therapeutic relevance of the PP2A-B55 inhibitory kinase MASTL/Greatwall in breast cancer by Mónica Álvarez-Fernández et al.

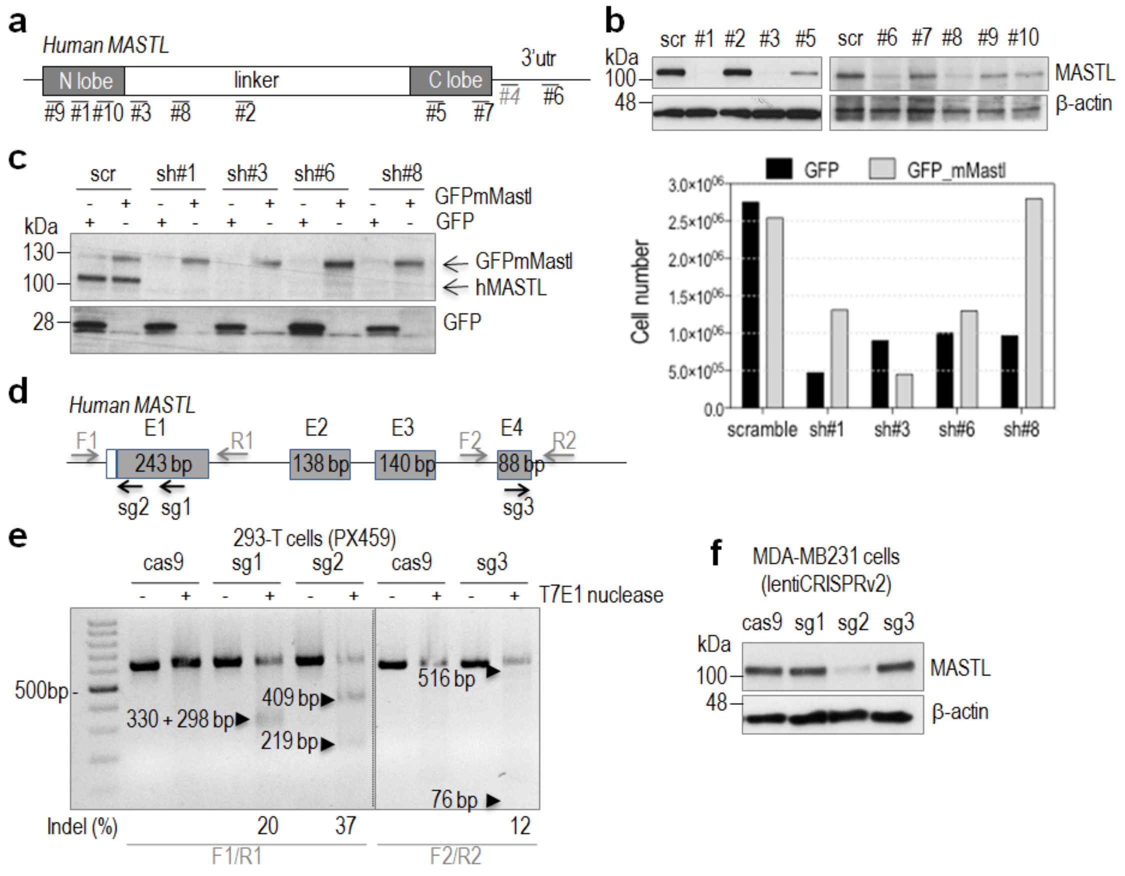

Figure S1. Selection of MASTL shRNAs and sgRNAs. (**a**) Schematic representation of human MASTL protein. Position of the shRNAs is indicated. (**b**) Immunoblotting analysis of MASTL protein levels in 293-T cells four days after infection with lentiviruses expressing the indicated shRNA sequences. (**c**) Rescue assay of shRNA-mediated MASTL depletion in cells expressing the mouse version of Mastl fused to GFP. Western-blot analysis of Mastl protein levels is shown in the left panel. The graph on the right panel represents the number of cells seven days after lentiviral infection. (**d**) Scheme of the first exons (1-4) of human MASTL gene. Positions of the sgRNAs and the primers used for PCR amplification are indicated. (**e**) T7E1 assay of MASTL locus. 293-T cells were transfected with a plasmid expressing Cas9 and the indicated sgRNAs, selected with puromycin for 48h, and collected 48h later for genomic DNA isolation. (**f**) Western-blot analysis of MASTL levels in MDA-MB-231 cells infected with lentiviruses expressing the indicated sgRNAs. Cell extracts were prepared 4 days after infection.

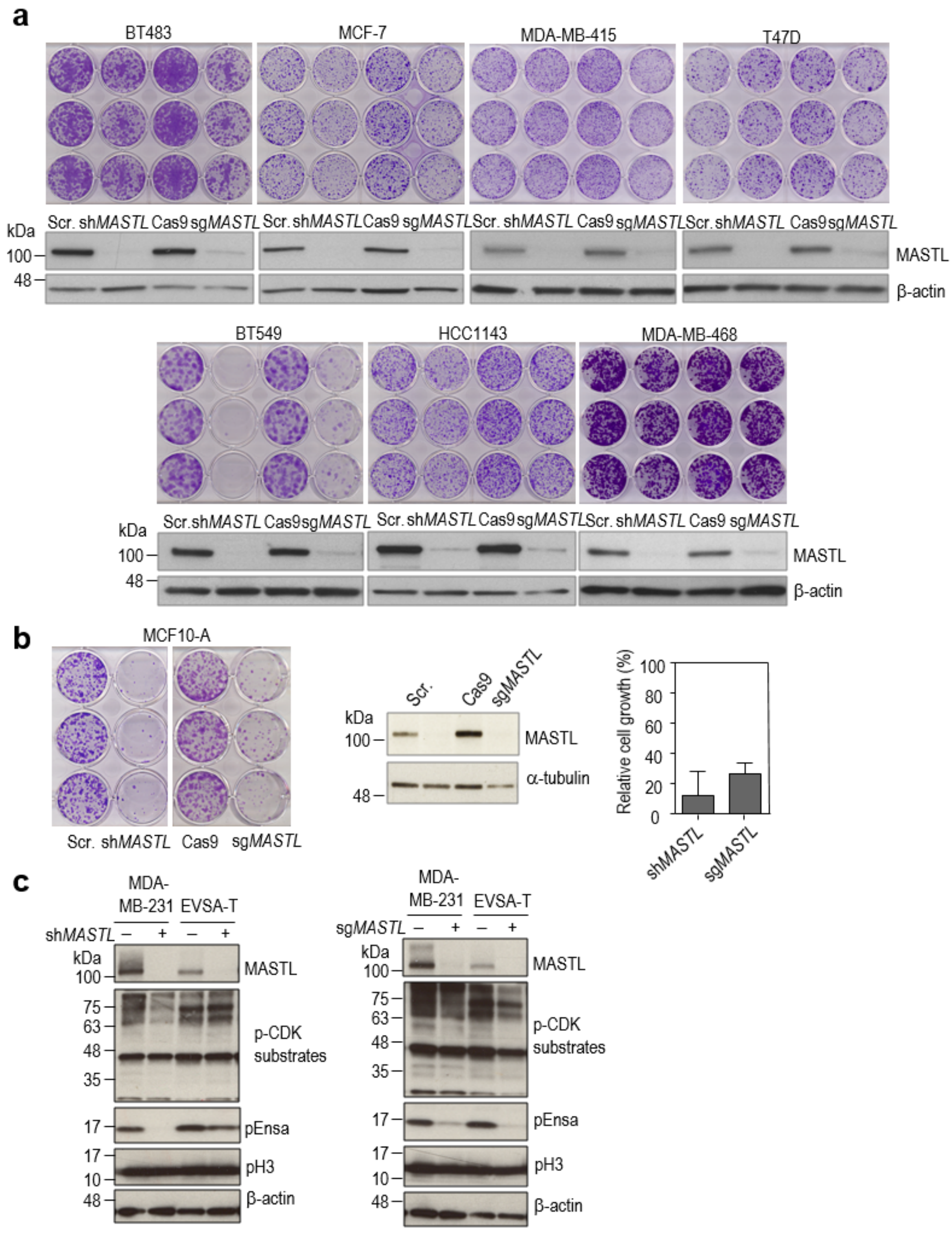

Figure S2. Effect of MASTL depletion in breast cell lines. (**a,b**) Representative colony formation assay after MASTL knockdown (shMASTL) or knockout (sgMASTL) in several hormone-positive (BT483, MCF-7, MDA-MB-415, T47D) and triple-negative/basal-like (BT549, HCC1143, MDA-MB-468) breast cancer cell lines and non-transformed MCF10-A cell line. Western-blot analysis of MASTL protein levels to monitor the efficiency of depletion is also shown. The graph in (b) shows mean + SD of three independent experiments. (**c**) Immunoblotting analysis of the phosphorylation levels of Ensa (MASTL substrate) and general CDK substrates in mitotic extracts of MASTL-depleted cells with either shRNAs (left panel) or sgRNAs and Cas9 (right panel). Two representative cell lines, one sensitive (MDA-MB-231) and one resistant (EVSA-T), are shown.

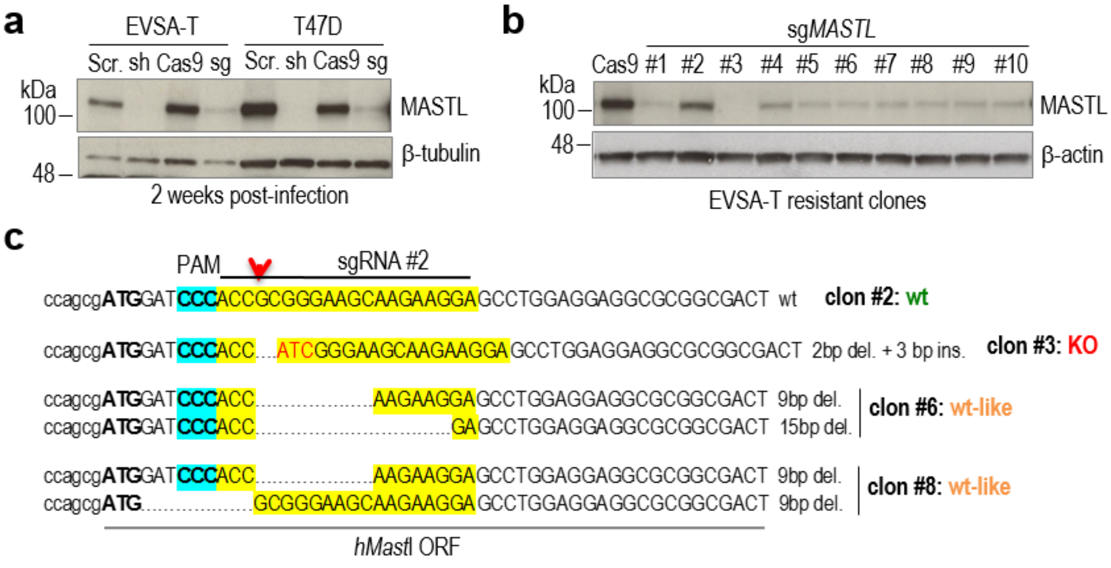

Figure S3. Analysis of survival clones in resistant breast cancer cell lines. (**a**) Western-blot analysis of MASTL protein levels after RNAi-mediated knockdown (sh) or CRISPR/Cas9-induced knockout (sg) in two resistant (EVSA-T, T47D) breast tumor cell lines at longer times (>2 weeks) after lentiviral infection. (**b**) Western-blot analysis of MASTL protein levels in ten EVSA-T surviving-resistant clones after Cas9/sg*MASTL* lentiviral infection. A Cas9 clone isolated from cells infected with empty vector only expressing Cas9 was used as a reference for MASTL wild-type protein expression. (**c**) Genomic analysis of some representative sg*MASTL* EVSA-T resistant clones. sgRNA and PAM sequence are highlighted in yellow and blue colors, respectively. Red arrowhead denotes predicted Cas9 cutting site. Note that, with the exception of clone #2 (wild-type) and clon #3 (knock-out), most clones express very low levels of MASTL protein (b), which correlate with the presence of in-frame indels (wt-like), as shown for clone #6 and clone #8. Wt, wild-type.

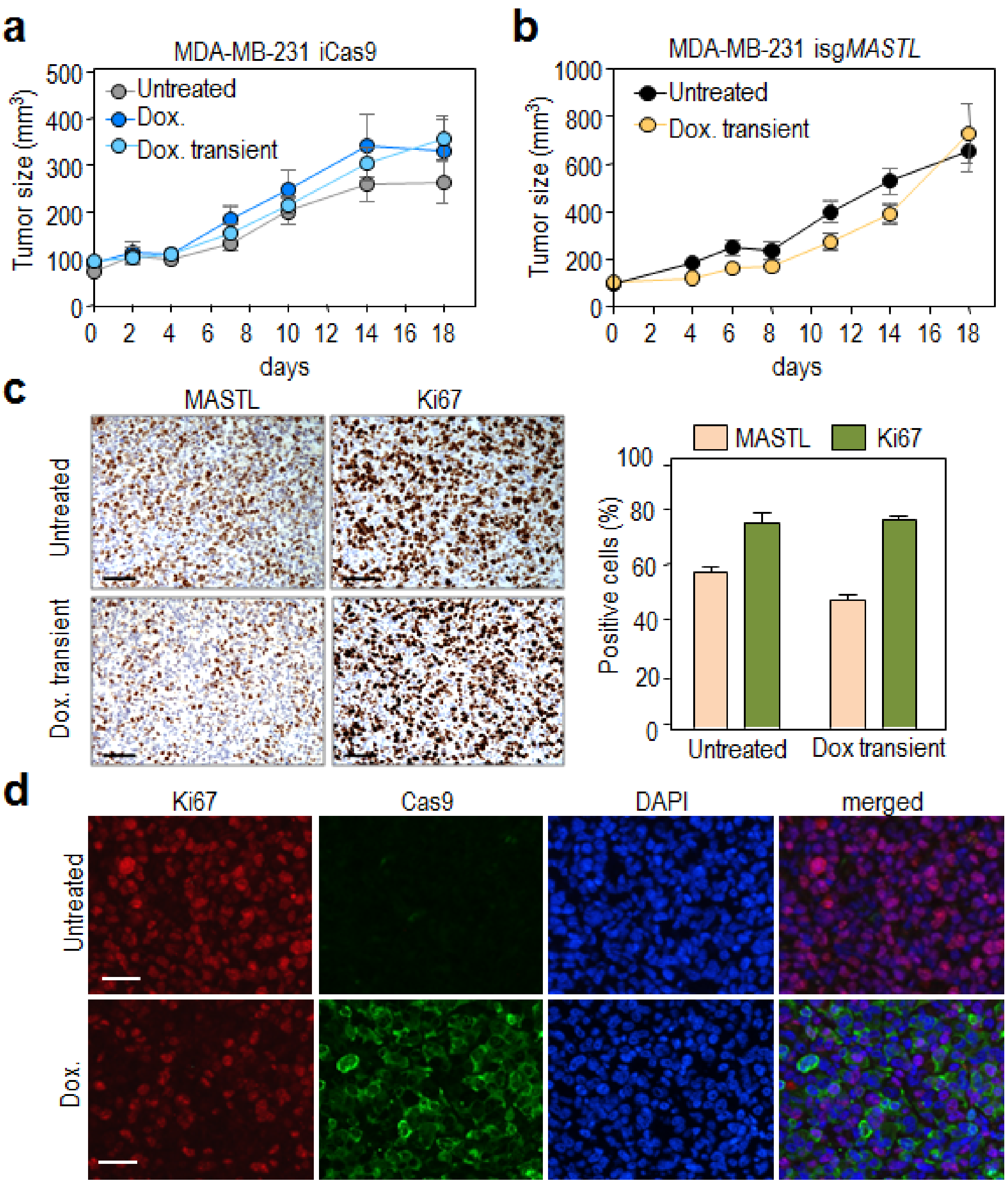

Figure S4. MASTL knockout impairs growth of tumor cells in vivo. (**a**) Growth of isgCas9 MDA-MB-231 xenotransplants either untreated (grey), after continuous treatment with doxycycline (dark blue) or after a transient (5 days) treatment with doxycycline (light blue). (**b**) Growth of isg*MASTL* MDA-MB-231 xenotransplants either untreated (black) or after a transient (6 days) treatment with doxycycline (yellow). (**c**) Immunohistochemical analysis of MASTL and Ki67 protein levels in representative tumors of the isg*MASTL* xenograft assay shown in (a). Bars represent mean + SEM of three independent tumors per experimental condition. (**d**) Immunocytochemical detection of Ki67 and Cas9 in representative tumors untreated or permanently treated with doxycycline as shown in Figure 5. Dox, doxycycline. Scale bars, 25 µm.

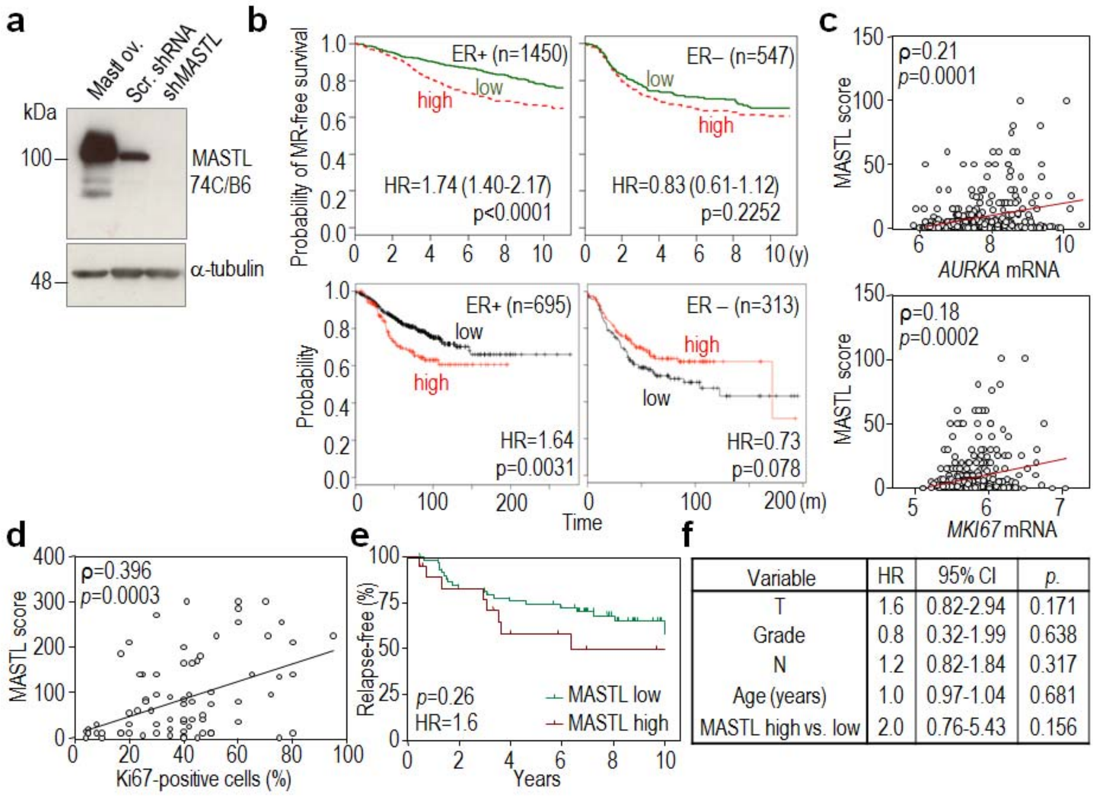

Figure S5. Extended analysis of MASTL expression in human breast tumors. (**a**) Western-blot showing the specificity of MASTL antibody. (**b**) Kaplan-Meier curve showing recurrence-free survival of estrogen receptor (ER)+ (left panel) or ER- (right panel) breast tumors (Gyorffy et al, 2010) with high or low *MASTL mRNA* expression using bc-GenExMiner (Jezequel et al, 2012)(upper panel) and Kaplan-Meier Plotter (lower panels)**.** (**c**) Correlation of MASTL protein expression levels with mRNA levels of *AURKA* (upper panel) or *MKI67* (lower panel) in the Metabric cohort. Statistical analysis was performed using the Spearman's test. (**d-f**) Analysis of MASTL protein expression in a triple-negative breast cancer (TNBC) cohort. (**d**) Mastl expression correlates with Ki67 protein levels (Spearman's test). (**e**) Kaplan-Meier plot for disease relapse, comparing high vs. low MASTL protein expression in the TNBC cohort of breast cancer patients. Statistical significance was calculated using the log-rank test. (**f**) Cox´s Proportionate Hazards Model, showing the risk variation attributable to each variable per unit increase. HR, Hazard ratio.

Supplementary Tables

Table S1. List of MASTL targeting sequences.

Name	Sequence
sh*MASTL* #1	CCATTCATTGTCCATTTGTA
sh*MASTL* #2	TCAGCCCTTAGATTCAGATA
sh*MASTL* #3	CTCTTGTGTAAACCTTGCTA
sh*MASTL* #5	CACAACAAGTATTCCAGAAT
sh*MASTL* #6	TGAAAGGAATATAGTCAGTA
sh*MASTL* #7	TTATCTGATAATGCTCAAAG
sh*MASTL* #8	AGTATAAGCATAACGAAATG
sh*MASTL* #9	TATGCAGTAAAGGTTGTTAA
sh*MASTL* #10	GTCAAGTCTCTCCTACATAT
sg*MASTL* #1	CCGAAGGCGCCCCGGCTAAT GGG
sg*MASTL* #2	TCCTTCTTGCTTCCCGCGGT GGG
sg*MASTL* #3	CATATTAAACTGACGGATTT TGG

Table S2. List and features of top-ranking off-target sites for sgMASTL in the human genome.

Sequence (20nt + PAM)*	Score	Mismatch position	locus	gene	Refseq	Clones analyzed	Detected changes
TCCTCCTGTCTTC CCGCGGT GGG	1.5	3MMs [5:8:9]	chr14:-103469840	CDC42BP	NM_006035	6	none
GCCCGCTTGCTT GCCGCGGT CGG	0.6	4MMs [1:4:5:13]	chr17:-42634850	FZD2	NM_001466	-	n.d.
ACCTTCTCCCTTC CCGGGGT TGG	0.4	4MMs [1:8:9:17]	chr17:-61494181	TANC2	NM_025185	6	none
TCTTTCTAGATTC CAGCGGT GGG	0.4	4MMs [3:8:10:15]	chr10:+118117785	CCDC172	NM_198515	6	none
TGCATCTTCCTTC CCGCGGG *CAG*	0.4	4MMs [2:4:9:20]	chr20:+9489644	LAMP5-AS1	NR_109957	6	none

* Mismatches are shown in red
n.d., not detected

Table S3. Hormone receptor positive breast cancer cohort (n=73).

**Characteristic**	**Value**
Age (median, STD)	52.8 (12.9)
T stage<br>T1<br>T2<br>T3<br>T4	 37 (50.7%)<br>26 (35.6%)<br>8 (11%)<br>2 (2.7%)
Grade<br>G1<br>G2<br>G3	 23 (31.5%)<br>34(46.6%)<br>16 (21.9%)
N stage<br>N0<br>N1<br>N2<br>N3	 26 (35.6%)<br>28 (38.4%)<br>9 (12.3%)<br>10 (13.7%)
Ki 67<br>High (>13%)<br>Low (<14%)	 38 (52.1%)<br>35 (47.9%)
Adjuvant chemoth.	58 (79.5%)
Adjuvant hormonotherapy	70 (96%)

Table S4. Triple-negative breast cancer cohort (n=84).

Characteristic	Value
Age (median, STD)	58.1 (11.5)
T stage<br>T1<br>T2<br>T3<br>T4	 25 (29.8%)<br>46 (54.8%)<br>10 (11.9%)<br>3 (3.6%)
Grade<br>G1<br>G2<br>G3<br>missing	 1(1.2%)<br>18 (21.4%)<br>48 (79.8%)<br>17 (20.2%)
N stage<br>N0<br>N1<br>N2<br>N3	 48 (57.1%)<br>16 (19%)<br>10 (11.9%)<br>10(11.9%)
Adjuvant chemoth.	74 (88%)
Adjuvant hormonetherapy	0 (0%)
